# Potential application of Russian Tarragon (*Artemisia dracunculus* L.) in health and sports

**DOI:** 10.1186/1550-2783-8-S1-P16

**Published:** 2011-11-07

**Authors:** Ivo Pischel, Natalie Burkard, Michaela Kauschka, Veronika Butterweck, Richard J Bloomer

**Affiliations:** 1 PhytoLab GmbH & Co. KG, Vestenbergsgreuth, Germany; 2 College of Pharmacy, Department of Pharmaceutics. University of Florida, Gainesville, USA; 3 Cardiorespiratory/Metabolic Laboratory, University of Memphis, Memphis, TN, USA

## Background

Tarragon is a spice herb with a long history of culinary and medical use. There exist two cultivars of this species: French Tarragon is used as a spice in cuisine and Russian Tarragon (RT) has been used medically in Russia and middle Asia, mainly to treat gastrointestinal disorders. However, recent studies also reported possible antidiabetic and hypoglycemic activities. Ribnicky*et al.* demonstrated that an ethanolic extract of RT was able to reduce blood glucose concentration in rodents. Tarragon like many other spices contains potential harmful essential oil constituents like estragole and methyleugenol. Thus, it was officially advised to limit the intake of such herbal spices. Therefore, as a solution to this problem, an aqueous extract of RT (RTE), which does not contain these compounds, was developed (Finzelberg GmbH & Co.KG, Germany) for further investigation. *In vivo* animal and human study demonstrate promising potential of the aqueous extract as a new potent antidiabetic agent.

## Methods

For the *in vivo* studies a standard animal model for testing of antidiabetic activity was used (non-fasted Wistar rats, n=8 / group). Blood glucose and insulin levels were determined with glucose challenge (2g/kg glucose infusion) and without (basal).

A randomized, double-blind, cross-over clinical trial in 12 non-diabetic men was performed to approve the effect of RT on serum glucose and insulin levels, as well as cardiovascular parameters. Subjects reported to the lab on 2 different mornings separated by 1 to 2 weeks, and ingested 75 g of dextrose in solution. 15 min before ingestion, men ingested either 2 g of RT or placebo. Blood samples were collected before ingestion of the RT and placebo, and several time points after dextrose administration.

## Results

It was shown that the aqueous extract of RT lowered the blood glucose level in both animals and humans (albeit non-statistically). The area under the blood glucose curve (AUC) was significantly decreased after oral administration of aqueous RTE to non-fasted Wistar rats (19,000 rel. AUC vs. 30,000 rel. AUC, n=8, p<0.001). For serum glucose, no condition (p=0.19) or condition x time (p=0.99) effect was noted in the clinical trial. Similar findings were noted for insulin. However, a time effect was noted (p<0.0001), with values at the 15 and 30 min blood collection times higher than pre-ingestion. Additionally, a potential positive impact of RTE administration on certain cardiovascular parameters was noted.

## Conclusion

The aqueous extract of RT is a promising and safe (lack of potentially harmful estragole and methyleugenol) ingredient for consideration in the development of functional foods or dietary and sports supplements with anti-hyperglycemic activity. In this context, a study investigating the potential of RT to increase serum insulin concentration while reducing blood glucose level for a given amount of glucose ingestion after an endurance exercise bout is ongoing. Thus, RT might also act as a “recovery agent”.

